# CEP55 Promotes Acral Melanoma Progression via MAPK Pathway and Predicts Survival Following Immunotherapy

**DOI:** 10.32604/or.2025.064780

**Published:** 2025-08-28

**Authors:** Meng Cao, Rundong Zhang, Anlan Hong, Shanyuan Ye, Zequn Qiu, Dongqing Li, Tong Lin, Yan Wang

**Affiliations:** Hospital for Skin Diseases, Institute of Dermatology, Chinese Academy of Medical Sciences & Peking Union Medical College, Nanjing, 210042, China

**Keywords:** Centrosomal protein 55 kDa (CEP55), acral melanoma (AM), mitogen-activated protein kinase (MAPK) pathway, immunotherapy

## Abstract

**Introduction:**

Acral melanoma (AM) is the predominant subtype of cutaneous melanoma in Asian populations, characterized by more aggressive clinical features and limited neoadjuvant therapy response. Centrosomal protein 55 kDa (CEP55) has been implicated in the pathogenesis of various malignancies, but its role in AM remains undefined.

**Methods:**

CEP55 expression in melanoma tissues and cell lines was analyzed by RT-qPCR, Western blotting, and immunohistochemistry (IHC). Databases (GEPIA, Sangerbox, Kaplan-Meier plotter, and TIMER) were used to analyze the expression of CEP55 and its correlation with clinical data of melanoma patients. Functional assays were conducted *in vitro* and *in vivo*. RNA sequencing (RNA-seq) and rescue experiments were used to explore underlying mechanisms. Tissue microarrays were used to verify the relationship between CEP55 and immune checkpoints.

**Results:**

CEP55 overexpression is associated with Breslow thickness and TNM stage in melanoma tissues and cell lines. CEP55 knockdown inhibited melanoma cell proliferation, migration, and invasion. And CEP55 activated mitogen-activated protein kinase (MAPK) signaling, leading to BRAF inhibitor resistance. Besides, CEP55 overexpression was associated with more favorable responses to immunotherapy in melanoma patients.

**Conclusions:**

CEP55 plays a critical role in AM progression and immunotherapy. Targeting CEP55 may be a promising therapeutic strategy for AM.

## 1 Introduction

Malignant melanoma is the leading cause of skin cancer-related mortality owing to its high metastatic potential and poor prognosis in advanced stages. Recent advancements in neoadjuvant therapies, such as immunotherapy and targeted therapies, have significantly improved survival rates of patients [1]. Among Asian populations, acral melanoma (AM) is the predominant subtype, whose primary lesions are observed frequently on extremities such as palms and soles [2,3]. The overall efficacy of targeted therapies remains suboptimal in Asia. This is largely because such mutations occur at relatively low frequencies in Asian patients with AM, and there is no specific treatment for AM patients without common driver mutations [4,5]. Studies showed that the subtypes of melanoma differed in regard to genetic mutations, treatment, and prognosis. Cyclin D1 (CCND1) and Neurofibromin 1 (NF1) are relatively frequent mutations in AM, and the BRAF mutation is at a low rate [6]. Previous studies have also suggested that AM shows less favorable responses to immunotherapy, potentially because of lower expression levels of immune checkpoint proteins, such as programmed cell death protein 1 (PD-1) [7,8]. The unsatisfactory prognosis of AM is possibly aggravated by an inadequate comprehension of its molecular mechanisms. The high mutation frequency of CCND1, cyclin-dependent kinase inhibitor 2A (CDKN2A), and other genes in AM suggests that centrosome aberrations caused by abnormal cell division and cell cycle disorders may be important mechanisms involved in AM [6,9].

CEP55 was believed to regulate cell exfoliation by recruiting endocytic transport complexes and served as a key mediator of cytokinesis [10,11]. CEP55 is predominantly expressed in the testis and thymus. The gene encoding CEP55 is located at 10q23.33 and produces a 464-amino-acid protein with three centrally positioned coiled-coil domains [10,12]. The abnormal expression of CEP55 may affect the completion of cell division, leading to the formation of multinucleated cells and chromosomal aneuploidy. This genomic instability provides a genetic basis for the accumulation of oncogenes and the deletion of tumor suppressor genes [13,14]. Various studies have confirmed that the high expression of CEP55 is often accompanied by the synergistic activation of other oncogenes, suggesting that it may jointly drive tumor progression through multi-molecular networks [15,16]. Alterations in CEP55 expression may result in the initiation of various diseases, some of which can be life-threatening [17,18]. Growing evidence implicates CEP55 in the pathogenesis of various cancers, such as ovarian cancer, renal cell carcinoma, and colorectal cancer [19–21]. In recent years, studies have found that CEP55 is abnormally overexpressed in various tumors and promotes tumor occurrence and development through mechanisms such as regulating cell proliferation, apoptosis, and metabolic reprogramming [22–24]. Furthermore, previous studies have indicated that CEP55-derived peptides exhibit significant therapeutic potential in cancers [25,26]. Besides, CEP55 participates in the remodeling of the tumor immune microenvironment and regulates the process of tumor immune response [21,27]. However, its role in melanoma is extremely limited.

Our previous study demonstrated that METTL3 exerted oncogenic functions in AM through m^6^A-dependent mechanism, and CEP55 may be a downstream target gene [28]. It prompted us to hypothesize that CEP55 may possess distinctive oncogenic mechanisms in AM. The PI3K/AKT signaling pathway, a downstream effector of CEP55, plays a pivotal role in melanoma pathogenesis [29,30], and CEP55 facilitates the progression of various tumors [19–21,26,31]. These findings indicate that CEP55 may be a significant tumor-promoting factor in AM. Considering the pivotal role of CEP55 in tumors, elucidating its influence on cellular processes such as proliferation, migration, and invasion could offer valuable insights for developing targeted therapeutic strategies in AM.

## 2 Materials and Methods

### 2.1 Tissue Samples Collection

The melanoma tumor and paratumor tissues used in this study were obtained from patients who underwent surgical treatment at our hospital. Following surgical resection, the two types of specimens were fixed in 4% paraformaldehyde (Biosharp, BL539A, Hefei, China), Nucleic Acid and Protein Stabilization Reagent (Beyotime, R0121, Shanghai, China), respectively. The samples were then processed into paraffin sections, and RNA and protein were extracted. The ethical committee of the Hospital for Skin Diseases, Institute of Dermatology, Chinese Academy of Medical Sciences & Peking Union Medical College approved melanoma tumor and paratumor tissues collection (Approval number: 2025-LC-001). And all informed consents had been signed. Tissue microarrays (Shanghai Zhuoli Biotech Company, Shanghai, China) were used to analyze the correlation between CEP55 and PD-L1. Tissue microarrays were approved by the Ethics Committee of Shanghai Zhuoli Biotech Company (Approval number: SHLLS-BA-22101102).

### 2.2 Cell Lines and Cell Culture

Primary human epidermal melanocytes (HEMa) were isolated from clinically obtained foreskin specimens following circumcision procedures in adults. The ethical committee of the Hospital for Skin Diseases, Institute of Dermatology, Chinese Academy of Medical Sciences & Peking Union Medical College approved tissues collection (Approval number: 2025-LC-001). And all informed consents had been signed. HEMa cultures were maintained in specialized melanocyte medium (MelM) (Sciencell, 2201, USA) containing 5% fetal bovine serum (FBS, Vivacell, C04001, Shanghai, China). Melanoma cell lines (A375, A2058, A875, SK-MEL-28, M14, MV3, HMY-1) were maintained by our research group. All these cells were propagated in high-glucose dulbecco’s modified eagle medium (DMEM) (Vivacell, Shanghai, China) enriched with 10% FBS. Besides, 1% penicillin-streptomycin antibody (Vivacell, C3420, Shanghai, China) and 0.1% mycoplasma removal reagent (CYTOCH, CM0002, Shanghai, China) were added to prevent microbial contamination. Cells were conducted under standard culture conditions (37°C, 5% CO_2_) with medium renewal at 48-h intervals and passaging at 80% confluence thresholds. All cell lines underwent periodic authentication through short tandem repeat profiling, with the latest verification completed in October 2023. Experimental protocols exclusively utilized low-passage cells (≤5 passages post-thaw) to ensure phenotypic stability.

### 2.3 Cell Transfection

In this study, complementary sense and antisense oligonucleotides were synthesized and cloned into GV493 vectors (hU6–MCS–CBh–gcGFP–IRES–puromycin) to construct lentivirus-carrying plasmids (shRNA) targeting CEP55. The NC sequence was TTCTCCGAACGTGTCACGT, and two sequences were designed for shCEP55: GAAGAGAAAGACGTATTGAAA (shCEP55-1) and CTGCTAAAGCAGCAAGAAGAA (shCEP55-2). All lentivirus vectors were constructed by GeneChem (Shanghai, China). Transfected cells were harvested with 2 μg/mL puromycin (Beyotime, ST551, Shanghai, China). Gene knockdown efficiency was verified using reverse transcription quantitative polymerase chain reaction (RT-qPCR) and western blotting (WB).

### 2.4 Western Blotting

Western Blotting was used to detect the expression level of protein. And proteins were lysed using radioimmunoprecipitation assay (RIPA) buffer (Beyotime, P0013C, Shanghai, China) supplemented with 10% protease inhibitors (cOmplete Tablets, Roche, 04693159001 Basel, Switzerland) and phosphatase inhibitors (PhosSTOP; Roche, 04906845001, Basel, Switzerland). Proteins were separated using a 10% or 4%–20% sodium dodecyl sulfate-polyacrylamide gel electrophoresis (SDS-PAGE) (Smart-lifesciences, SLE015/SLE017, Changzhou, China) in a vertical electrophoresis apparatus at 120 V for 60 min and transferred onto polyvinylidene difluoride (PVDF) membranes (Millipore, IPVH00010, USA). NcmBlot blocking buffer (New Cell & Molecular Biotech Co., Ltd., P30500, China) was used to incubate the membranes for 30 min at room temperature. The following antibodies were used: CEP55 (1:20,000, Proteintech, 23891-1-AP, Wuhan, China), glyceraldehyde 3-phosphate dehydrogenase (GAPDH) (1:10,000, ab181602, Abcam, ab181602, USA), E-cadherin (1:5000, Proteintech, 20874-1-AP, Proteintech, Wuhan, China), N-cadherin (1:1000, 22018-1-AP, Proteintech, 22018-1-AP, Wuhan, Wuhan, China), extracellular signal-regulated kinase **(**ERK) 1/2, (1:2000, Cell Signaling Technology, #4695, USA), p38 (1:1000, Cell Signaling Technology, #8690, USA), p-ERK1/2 (1:1000, Cell Signaling Technology, #4370, USA) and p-p38 (1:1000, Cell Signaling Technology, #4511, USA). All antibodies were incubated at 4°C overnight. The secondary antibody (Goat Anti-Rabbit IgG H&L (HRP), 1:20000, Abcam, ab205718, USA) was incubated for 1 h at room temperature. Protein detection was performed using an electrochemiluminescence system (Amersham ImageQuant 800, Cytiva, USA).

### 2.5 qRT-PCR (Quantitative Reverse Transcription PCR)

We performed qRT-PCR to evaluate gene expression levels in AM tissues and cell lines. Total RNA was extracted from various samples using TRIzol reagent (Invitrogen, 15596018CN, USA) following the manufacturer’s protocol. RNA samples (500–1000 ng) were assessed for purity and concentration and then reverse-transcribed using the PrimeScript™ RT Master Mix (Accurate Biotechnology, AG11728, Hunan, China). Quantification of gene expression was performed using qPCR on an LC480 system (Roche Applied Science, Mannheim, Germany) with SYBR Master Mix (Accurate Biotechnology, AG11701, Hunan, China). The reaction conditions were set according to the instructions of the kit. The expression data were normalized to that of GAPDH, and relative CEP55/GAPDH mRNA expression was calculated using the 2^−△△Ct^ method. The primer (Species: *Homo sapiens*) sequences were as follows: GAPDH: Primer-F: 5^′^-TGACTTCAACAGCGACACCCA-3^′^, Primer-R: 5^′^-CACCCTGTTGCTGTAGCCAAA-3^′^, CEP55: Primer-F: 5^′^-GACCGTTGTCTCTTCGATCGCTTC-3^′^, Primer-R: 5^′^-GTATTCCACATGGACAAGCAGATC-3^′^.

### 2.6 Immunohistochemistry Staining (IHC)

IHC staining was performed using the following antibodies: anti-CEP55 (1:1000; Proteintech, 23891-1-AP), PD-L1 (1:1000; Proteintech, 66248-1-Ig), and anti-Ki-67 (1:400; Abcam, ab16667). Tissues were embedded in paraffin and sectioned into 4-µm slices. After deparaffinization with xylene (Sinopharm, Shanghai, China) and Gradient concentration ethanol (Sinopharm, Shanghai, China), antigen retrieval was performed in EDTA buffer (Recordbio, RC04, Shanghai, China) using microwave irradiation. Endogenous peroxidase activity was blocked with 3% H_2_O_2_ (Sinopharm, 7722-84-1, Shanghai, China) for 25 min, followed by 3% Bovine Serum Albumin (BSA) (Beyotime, ST023, Shanghai, China) blocking. Sections were incubated with primary antibody at 4°C overnight, then treated with HRP-conjugated secondary antibody (Recordbio, RCA054) for 1 h at room temperature. Diaminobenzidine (DAB) (Recordbio, RCD002) and hematoxylin (Recordbio, RC0060) were used to visualize the staining and counterstain the slides. Hematoxylin-stained nuclei appeared blue, whereas DAB-positive nuclei appeared brownish yellow. Images of tissue microarrays were captured using a microscope (KF-PRO-005-EX, KFBIO, Zhejiang, China). Samples in tissue microarrays without tumors, defects, or artifacts were excluded from the analysis. The remaining images were captured using an inverted microscope (Nikon, EclipseTi, Tokyo, Japan).

### 2.7 Detection of Cell Proliferation and Cell Viability by CCK8 Assay

In the cell proliferation assay, cell groups were seeded into 96-well plates at a density of 2000 cells per well. At 24–96 h, 10 μL of Cell Counting Kit-8 (CCK8) reagent (Abbkine, BMU106, Wuhan, China) was added to each well. After incubating at 37°C for 2 h, absorbance at 450 nm was measured using a microplate reader (Thermo Fisher Scientific, Multiskan Spectrum, USA). The cells were cultured for 4 days, and a growth curve was plotted based on the measured optical density values. In the cell viability assay, cell groups were seeded into 96-well plates at a density of 5000 cells per well. The cells were cultured with different concentrations of BRAF inhibitor (Vemurafenib, MCE, HY-12057, Shanghai, China) for 48 h. And CCK8 was added with the same volume in cell proliferation, and OD450 nm was detected at 48 h. Cell viability = (experimental group OD450 nm − blank group OD450 nm)/(control group OD450 nm − blank group OD450 nm) × 100%.

### 2.8 Scratch Assay

The scratch assay was used to assess cell migration and wound-healing ability. In a 6-well plate, 5 × 10^5^ cells were seeded per well and cultured for 24 h to achieve 90%–95% confluence. A linear wound was created using a 200-μL pipette tip. Cells were then cultured in serum-free DMEM, and images were captured to measure the wound gap, tracking the dynamics of cell migration and wound healing. The wound area migration ratio was used to evaluate cell migration ability.

### 2.9 5-Ethynyl-2^′^-Deoxyuridine (EdU) Assay

Cells were seeded into a 96-well plate at a density of 3 × 10^3^ cells per well. The following day, the cells were incubated with 10 μM EdU (Abbkine, KTA2031, Wuhan, China) for 2.5 h at 37°C. Subsequently, they were fixed with 4% paraformaldehyde and permeabilized with 0.5 %TritonX-100 (Beyotime, ST1723, Shanghai, China). The cells were then stained with AbFluor545 dye solution and Hoechst 33342 (Abbkine, KTA2031, Wuhan, China). Imaging was performed in three randomly selected fields using an inverted fluorescence microscope (Nikon, EclipseTi, Tokyo, Japan). The percentage of EdU-positive cells was calculated as (number of EdU-positive cells/number of Hoechst-positive cells) × 100%.

### 2.10 Colony Formation Assay

Logarithmic-phase cells were seeded into six-well plates at a density of 2000 cells per well. After culturing for 10 days at 37°C, the cells were fixed with paraformaldehyde for 20 min and stained with crystal violet for 20 min. Colony numbers were analyzed using ImageJ software (Version 2, NIH, USA).

### 2.11 Migration and Invasion Analysis

The Transwell assay was utilized to assess cell migration and invasion ability in melanoma cell lines. In the invasion experiment, cells were plated in inserts upper chamber (Corning, New York, NY, USA), precoated with 50 μL of Matrigel (Nest, 211212, Wuxi, China). For migration evaluation, uncoated chambers were employed. The cells were cultured in serum-free DMEM for 24 h at a density of 1 × 10^5^ cells/mL. The upper chamber was filled with 200 μL of serum-free DMEM, whereas the lower chamber contained 600 μL of high-glucose DMEM supplemented with 10% fetal bovine serum. Following a 24-h culture period under standard conditions (37°C, 5% CO_2_), non-migratory cells were removed from the membrane’s apical side. Cells remaining on the lower surface were fixed with 4% paraformaldehyde for 30 min and stained with 0.1% crystal violet for 30 min. The image was captured using an inverted microscope (Nikon, EclipseTi, Tokyo, Japan).

### 2.12 *In Vivo* Xenograft Experiment

A cohort of eighteen female BALB/c nude mice (age: 6 weeks old, weight: 18–20 g) was acquired from GemPharmatech (Nanjing, China). The animals were maintained in a specific pathogen–free environment under standard laboratory conditions with free access to autoclaved feed and water. They were randomly divided into three groups. All animal experiments were approved by Institutional Animal Care and Use Committee of Hospital for Skin Diseases, Institute of Dermatology, Chinese Academy of Medical Sciences & Peking Union Medical (Approval number: 2023-DW-013), and all procedures complied with the National Institutes of Health (NIH) Guide for the Care and Use of Laboratory Animals or equivalent guidelines. Subcutaneous xenograft establishment involved injecting 2 × 10^6^ A2058 cells (NC, shCEP55-1, or shCEP55-2) into the right posterior femoral region. Tumor size was measured using a Vernier caliper (Greener, China) every two days. Tumor volume = (Length × Width^2^)/2. And tumor weight was measured by balance. Tumors were collected and fixed in 4% paraformaldehyde, Nucleic Acid and Protein Stabilization Reagent, respectively, for further analysis.

### 2.13 Bioinformatics Analysis

RNA seq procedures commenced with qualitative assessment of nucleic acid samples using spectrophotometric quantification (NanoDrop 2000, Thermo Fisher Scientific). Subsequent library preparation and high-throughput sequencing were performed through collaboration with Hongsun Biotechnology Co. (Shanghai, China). Using RNA-seq data, we conducted gene ontology (GO) analysis in the R programming environment (Version 2.0) with the GO package to examine the biological processes, cellular components, and molecular functions associated with differentially expressed mRNAs. Pathway analysis was subsequently performed using the Kyoto Encyclopedia of Genes and Genomes (KEGG) database. Furthermore, we analyzed the differential expression of CEP55 and its correlation with immunotherapy through the gene expression profiling interactive analysis (GEPIA), SangerBox 3.0, Kaplan-Meier Plotter, and Tumor Immune Estimation Resource (TIMER) databases [32–36].

### 2.14 Statistical Analysis

The Data are presented as mean ± SD from three independent experiments (*n* = 3). Statistical analysis was performed using Statistical Package for the Social Sciences (SPSS) 23.0 software (SPSS, Inc., Chicago, IL, USA) and GraphPad Prism 8 (version 8.0.0 for Windows, GraphPad Software, San Diego, CA, USA). Variables were tested using a two-tailed Student’s *t*-test and the χ2 test, as appropriate. One-way ANOVA or two-way ANOVA was used for comparisons among multiple groups. Pearson correlation analysis was used to determine the relationship between PD-L1 and CEP55 expression levels. Statistical significance was defined as *p < 0.05, **p < 0.01, ***p < 0.001, and ****p < 0. 0001.

## 3 Results

### 3.1 CEP55 Upregulation in Melanoma Cell Lines and Tissues

**Figure 1:**
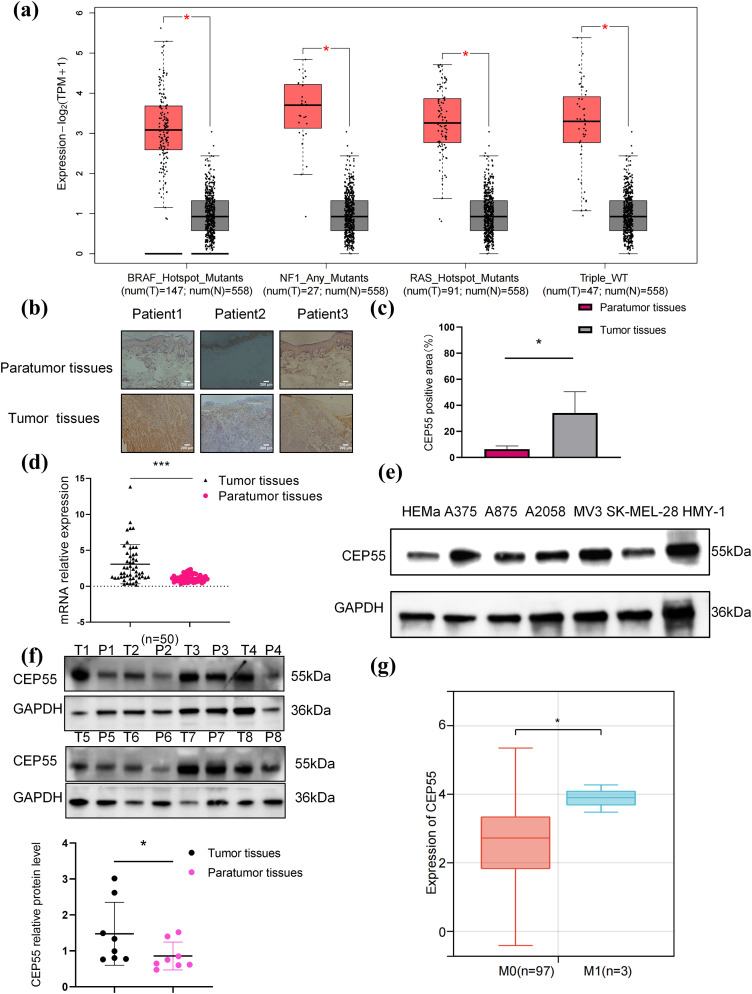
CEP55 was overexpressed in melanoma. **(a)** CEP55 expression levels in melanoma tissues with various gene mutations. **(b, c)** Immunohistochemical staining showing CEP55 expression in melanoma and paratumor tissues, scale bar = 200 μm. **(d)** RT-qPCR results showing CEP55 RNA expression levels in 50 pairs of tumor and paratumor tissues. **(e)** CEP55 expression levels in various melanoma cell lines detected by RT-qPCR. **(f)** Western blot analysis of CEP55 protein levels in 8 pairs of melanoma tumor (T) and paratumor (P) tissues. **(g)** Correlation between CEP55 expression and clinical M stage of melanoma analyzed using SangerBox 3.0 (*p < 0.05, ***p < 0.001)

Using the GEPIA platform, we analyzed the data of patients with melanoma and normal skin tissues from the TCGA and GTEx databases. The analysis demonstrated that CEP55 expression levels were significantly higher in tumor tissues with various gene mutations than in normal tissues (Fig. 1a). IHC staining further confirmed this finding, showing that CEP55 expression was upregulated in melanoma (Fig. 1b,c). To further assess CEP55 expression in AM, we performed qRT-PCR and WB to quantify CEP55 levels in melanoma tissue samples and cell lines. In 50 pairs of tumor and paratumor tissues, CEP55 RNA expression (Fig. 1d) was markedly elevated in melanoma tissues. WB analysis also verified increased CEP55 expression in 6 melanoma cell lines compared to human primary melanocytes (Fig. 1e). Moreover, WB confirmed elevated CEP55 expression in tumor tissues (Fig. 1f). These findings indicated that CEP55 may contribute to melanoma development and progression.

### 3.2 Increased Expression of CEP55 Is Associated with Poor Prognosis in Melanoma

By using Sangerbox, we found a significant correlation between CEP55 expression levels and tumor M stage (AJCC TNM stage, M0 = without metastasis, M1 = with metastasis) stage (Fig. 1g). To further explore the role of CEP55 expression in the clinical diagnosis and prognosis of AM, patients were divided into high- and low-expression groups based on the median PCR results of 50 patients. The chi-square test analysis demonstrated a significant correlation between CEP55 expression levels and breslow thickness. Meanwhile, the expression level of CEP55 was also significantly correlated with the tumor TNM stage (Table 1). Greater thickness and advanced stage indicate poorer survival outcomes, so these findings indicate that high CEP55 expression is linked to a worse prognosis in patients with melanoma.

**Table 1:** RNA expression level of CEP55 and the clinicopathological features of melanoma patients (*n* = 50) (*p < 0.05)

Characteristics	Number	CEP55 Expression		*p* Value
		High	Low	
**All patient**	50	25	25	
**Age (years)**				
≥60	28	14	14	1
<60	22	11	11	
**Sex**				
Male	19	11	8	0.567
Female	31	14	17	
**Tumor size (cm)**				
≥3	23	13	10	0.777
<3	27	12	15	
**Breslow thickness (mm)**				
≥1	39	23	16	0.017*
<1	11	2	9	
**Ulcer or erosion**				
Yes	13	7	6	0.747
No	37	18	19	
**TNM stage**				
I–II	38	16	22	0.047*
III–IV	12	9	3	
**Tumor metastasis or recurrence**				
Yes	14	5	9	0.208
No	36	20	16	

### 3.3 CEP55 Regulates Melanoma Proliferation In Vitro

**Figure 2:**
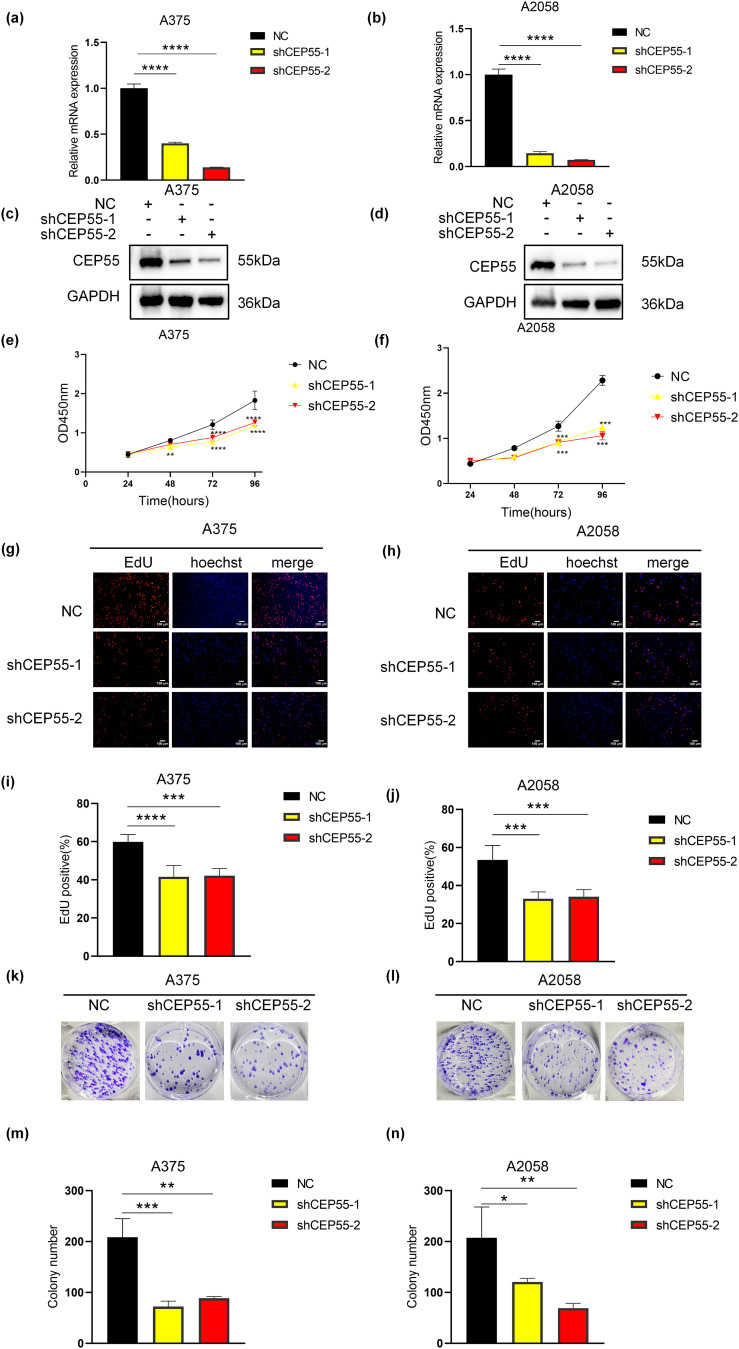
CEP55 downregulation inhibited melanoma cell proliferation *in vitro*. **(a, b)** Successful construction of NC, shCEP55-1, and shCEP55-2 in A375 and A2058 melanoma cell lines confirmed by RT-qPCR. **(c, d)** CEP55 protein levels confirming knockdown efficiency in A375 and A2058 cells via western blot analysis. **(e, f)** Cell proliferation was assessed using the CCK8 assay at 24–96 h. **(g, h)** EdU assay showing decreased proliferation in shCEP55-1 and shCEP55-2 groups compared with that in the NC group, Scale bar = 100 μm. **(i, j)** Quantification of EdU-positive cells showed a significant reduction in the shCEP55-1 and shCEP55-2 groups compared to NC. **(k, l)** Colony formation assay performed in melanoma cell lines. **(m, n)** Quantification of colonies showed significant reductions in colony numbers in the shCEP55-1 and shCEP55-2 groups. Each experiment was performed in triplicate. Data are presented as the mean ± standard deviation (SD). *p < 0.05, **p < 0.01, ***p < 0.001 and ****p < 0.0001

To investigate the functional role of CEP55 in melanoma, we silenced its expression in A375 and A2058 melanoma cell lines by transfecting with lentivirus-derived shRNAs (shCEP55-1 and shCEP55-2, respectively). This knockdown significantly reduced CEP55 expression compared with that in nontargeted control (NC) cells, as validated by qRT-PCR (Fig. 2a,b) and western blotting (Fig. 2c,d). We next assessed the effects of CEP55 knockdown on the biological functions of A375 and A2058 cells. CCK-8 assays revealed that reducing CEP55 expression markedly suppressed melanoma cell proliferation (Fig. 2e,f). To further examine the effects of CEP55 on cell proliferation, EdU assays (Fig. 2g,h) were performed. In the EdU assays, the proportion of EdU-positive cells in the shCEP55-1 and shCEP55-2 groups was significantly lower than that in the NC group, a finding consistent across A375 (Fig. 2i) and A2058 (Fig. 2j) cell lines. Similarly, in colony formation assays (Fig. 2k,l), the number of colonies formed by shCEP55-1 and shCEP55-2 cells was significantly lower than that of NC cells in the A375 (Fig. 2m) and A2058 (Fig. 2n) cell lines. Collectively, these results indicated that reducing CEP55 expression significantly diminished the proliferation capacity of melanoma cells. These complementary assays collectively established CEP55 as a critical regulator of melanoma proliferative capacity.

### 3.4 CEP55 Downregulation Inhibits Melanoma Cell Migration and Invasion In Vitro

**Figure 3:**
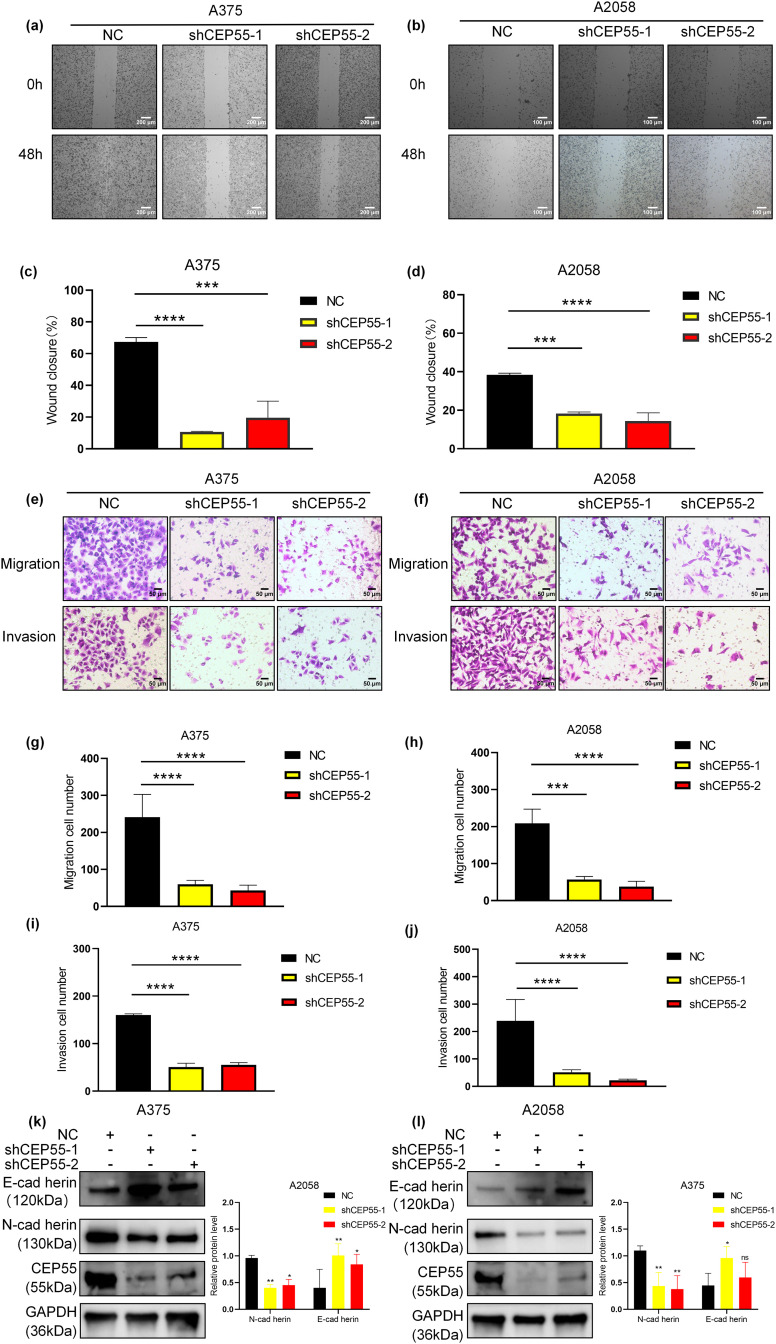
CEP55 downregulation inhibits melanoma cell migration and invasion of melanoma *in vitro*. **(a)** Wound-healing (scratch) assays were conducted over 48 h in A375, Scale bar = 200 μm. **(b)** Wound-healing (scratch) assays were conducted over 48 h in A2058, Scale bar = 100 μm. **(c, d)** Quantification of wound closure showing significantly reduced migration in shCEP55-1 and shCEP55-2 groups. **(e, f)** Transwell assays (with and without Martigel) were performed to assess change in tumor cell migration and invasion. Scale bar = 50 μm. **(g–j)** Quantification of migrated and invaded cells shows significant decreases in the shCEP55-1 and shCEP55-2 groups compared to NC. **(k, l)** WB showed decreased N-cadherin and increased E-cadherin protein levels in CEP55 knockdown groups. *p < 0.05, **p < 0.01, ***p < 0.001 and ****p < 0.0001, ns *p* ≥ 0.05

Building upon the clinical correlation between elevated CEP55 expression and melanoma progression in advanced M stage and increased Breslow thickness, we compared the migration and invasion capabilities of NC, shCEP55-1, and shCEP55-2 cells. The scratch test revealed that the wound closure area of shCEP55-1 and shCEP55-2 cells was significantly smaller than that of NC cells at 48 h after wound formation (Fig. 3a–d). It demonstrated markedly impaired migration capacity in CEP55-depleted cells. Transwell assays, both with and without Matrigel, were performed to assess cell invasion and migration capabilities, respectively (Fig. 3e,f). Transwell assays without Matrigel showed a significant reduction in the number of migration cells (Fig. 3g,h), whereas assays with Matrigel demonstrated a marked decrease in the number of invasion cells (Fig. 3i,j). WB analysis indicated a reduction in N-cadherin protein levels and an increase in E-cadherin protein levels, reflecting changes in the epithelial-mesenchymal transition (EMT) process (Fig. 3k,l). Collectively, these findings demonstrated that CEP55 knockdown significantly impaired the migration and invasion abilities of melanoma cells *in vitro*.

### 3.5 CEP55 Promotes Melanoma Tumor Growth In Vivo

**Figure 4:**
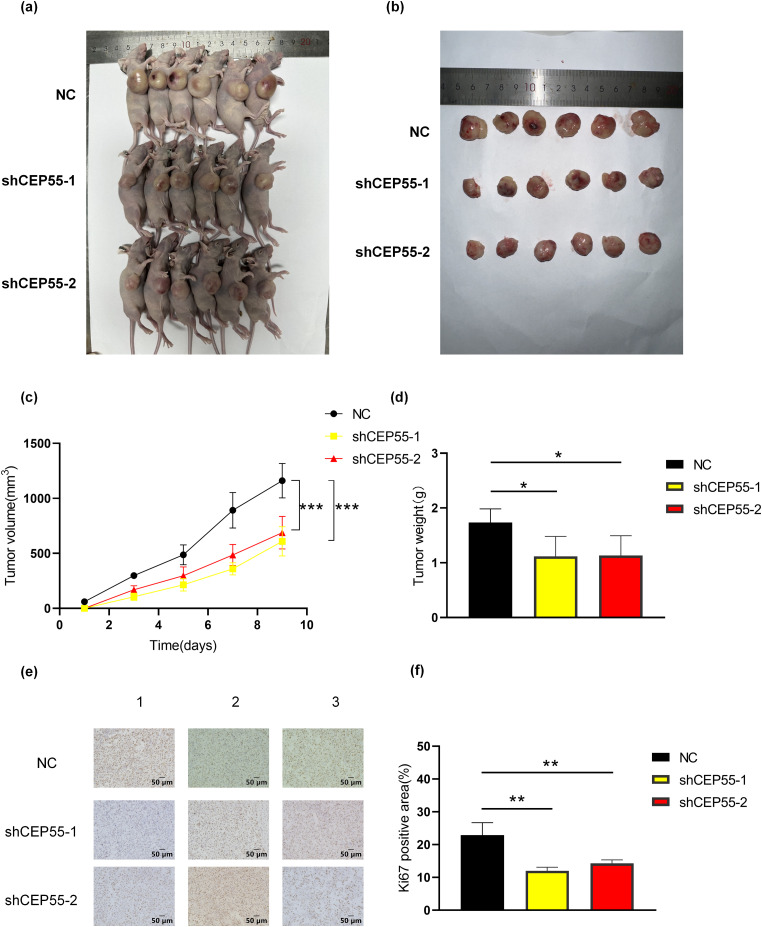
Downregulation of expression levels inhibits melanoma cell growth *in vivo*. **(a, b)** Representative images of tumors from melanoma xenograft models 9 days after inoculation. **(c)** Based on the volume equation, a growth curve for the tumor was drawn. **(d)** Tumor weights were measured after excision from the mice. **(e, f)** Representative images of Ki67 immunohistochemical staining in xenograft tumor sections and quantification of Ki67-positive area are shown (scale bar = 50 μm) (*p < 0.05, **p < 0.01, ***p < 0.001)

To validate the functional role of CEP55 *in vivo*, A2058 shCEP55-1, A2058 shCEP55-2, and A2058-NC cells were implanted into the flanks of nude mice, establishing three groups of xenograft models (Fig. 4a). Tumors were excised on day 9 post-implantation (Fig. 4b). In both groups with reduced CEP55 expression (A2058 shCEP55-1 and A2058 shCEP55-2), tumor volume and weight were significantly lower than those in the NC group (Fig. 4c,d). IHC staining further confirmed reduced expression of the melanoma proliferation marker Ki-67 in the shCEP55 groups (Fig. 4e,f). These findings demonstrated that CEP55 knockdown suppressed melanoma proliferation *in vivo*.

### 3.6 CEP55 Regulates Downstream Gene Transcription Expression

**Figure 5:**
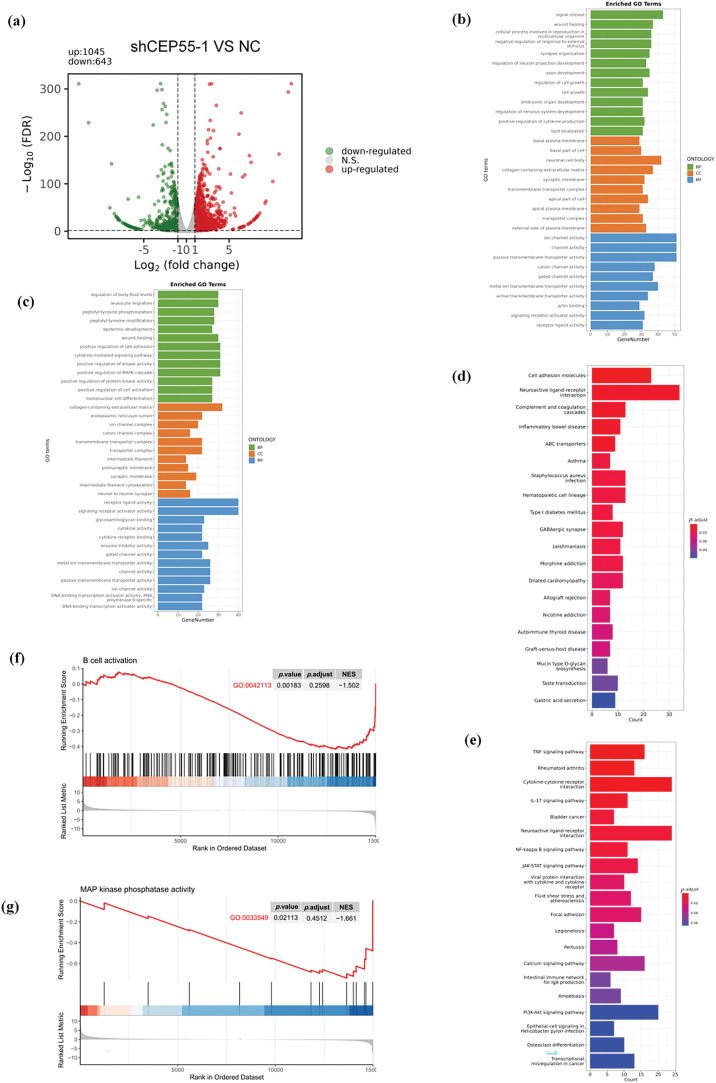
Regulation of CEP55 on the transcriptome profile in melanoma cells. **(a)** Volcano plot showing DEGs identified by RNA-seq between the NC and shCEP55 groups in A2058 cells. **(b, c)** GO analysis of significantly upregulated and downregulated DEGs identified via RNA-seq. **(d, e)** KEGG analysis of significantly upregulated and downregulated DEGs identified via RNA-seq. **(f)** GSEA indicates the involvement of DEGs in B cells. **(g)** GSEA indicates the involvement of DEGs in the MAPK pathway

To delineate CEP55’s transcriptomic regulatory network in melanoma progression, we conducted RNA-seq via the Illumina NovaSeq 6000 platform on A2058 shCEP55-1 and A2058 NC cells. Following CEP55 downregulation, differential expression analysis identified 1688 significantly differentially expressed genes (DEGs) globally (*p* < 0.05 and |log_2_FoldChange| ≥ 1). 1045 DEGs were found upregulated and 643 were found downregulated (Fig. 5a). Gene Ontology (GO) analysis revealed that upregulated DEGs were primarily associated with signal release, basal plasma membrane, and ion channel activity (Fig. 5b). In contrast, downregulated DEGs were associated with the regulation of body fluid levels, collagen-containing extracellular matrix, and receptor–ligand activity (Fig. 5c). Kyoto Encyclopedia of Genes and Genomes (KEGG) analysis indicated that the top three pathways enriched in upregulated DEGs are cell adhesion molecules, neuroactive ligand-receptor interaction, complement, and coagulation cascades (Fig. 5d). The top three pathways enriched in downregulated DEGs are the TNF signaling pathway, rheumatoid arthritis, and cytokine-cytokine receptor interaction (Fig. 5e). Notably, B cell-related signaling pathways were significantly enriched following CEP55 knockdown, indicating that CEP55 may influence the immune response in melanoma (Fig. 5f). Additionally, the mitogen-activated protein kinase (MAPK) signaling pathway was significantly enriched, indicating that CEP55 may regulate this pathway to affect melanoma progression (Fig. 5g). This observation was consistent with findings on CEP55 in bladder cancer [37]. However, it remains uncertain whether CEP55 affects melanoma through the MAPK signaling pathway.

### 3.7 CEP55 Regulates ERK1/2 and p38 MAPK Pathways in Melanoma

**Figure 6:**
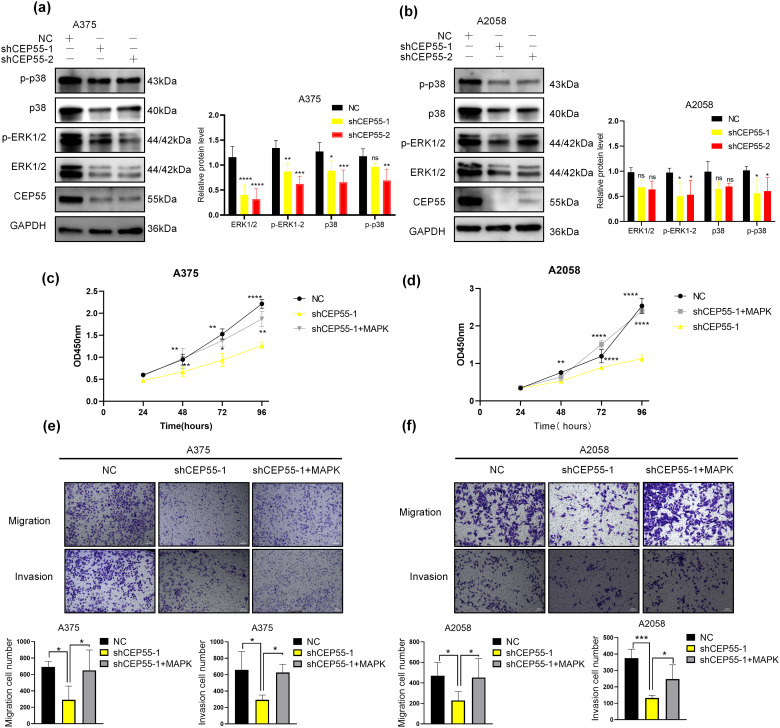

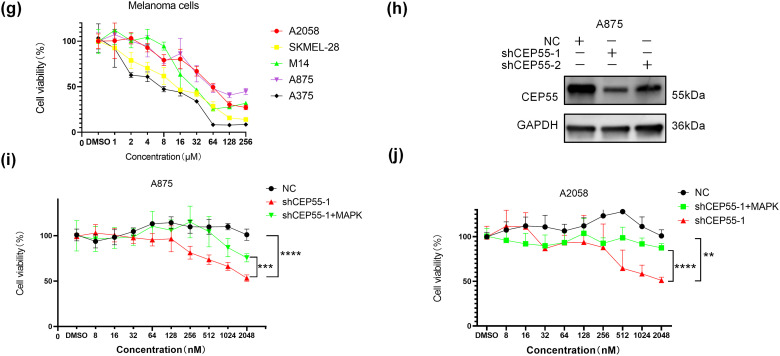
CEP55 promotes melanoma development and drug resistance by activating the ERK1/2 and p38 MAPK pathways. **(a, b)** The expression levels of ERK1/2, p-ERK1/2, p38, and p-p38 decreased in different degree after the downregulation of CEP55. **(c, d)** CCK8 assays revealed a partial restoration of cellular proliferative capacity upon the addition of a MAPK pathway activator. **(e, f)** Transwell assays demonstrated partial restoration of cell invasion and migration abilities following MAPK activator treatment, scale bar = 100 μm. **(g)** Cell viability assessments across various drug concentrations in melanoma cell lines (A375, A2058, A875, M14, and SKMEL28) identified A2058 and A875 as more drug-resistant cell lines. **(h)** A CEP55 knockdown A875 cell line was successfully established, with WB confirming knockdown efficiency. **(i, j)** CEP55 knockdown increased melanoma cell sensitivity to vemurafenib; however, the resistance partially re-emerged upon the addition of MAPK pathway activators. (ns *p* ≥ 0.05, *p < 0.05, **p < 0.01, ***p < 0.001, ****p < 0.0001)

To verify the effect of CEP55 on the MAPK signaling pathway in melanoma, we conducted WB analysis. Following the CEP55 knockdown, the protein levels of downstream targets such as total ERK1/2 and total p38 were reduced in different degree. There was a significant difference in A375 but not in A2058. Moreover, the levels of phosphorylated forms p-erk1/2 and p-p38 were significantly decreased (Fig. 6a,b). These results indicated that CEP55 may affect the biological function of melanoma cells by affecting the phosphorylation of p38 and erk1/2 in the MAPK signaling pathway. To further confirm this conclusion, we performed rescue experiments. The addition of vacquinol-1 (MAPK activator) to shCEP55-1 cells reactivated MAPK signaling. The group treated with vacquinol-1 was named as shCEP55-1 + MAPK. Cells of the shCEP55-1 + MAPK group were cultured in a medium with 10 μM Vacquinol-1 for 24 h before experiments. The CCK8 assay showed that the shCEP55-1 + MAPK group exhibited significantly enhanced proliferation compared with that of the shCEP55-1 group, indicating that the activation of MAPK signaling could partially rescue the suppressed proliferation caused by CEP55 knockdown (Fig. 6c,d). Similarly, the invasion and migration abilities of A375 and A2058 cells in the shCEP55-1 + MAPK group were also significantly increased compared with those in the shCEP55-1 group (Fig. 6e,f). These findings further supported our conclusion that CEP55 regulated the proliferation, invasion, and migration of melanoma cells through the MAPK signaling pathway.

### 3.8 CEP55 Induces Drug Resistance to BRAF Inhibitors (BRAFi) in Melanoma

Reactivation of the MAPK signaling pathway is a key mechanism underlying resistance to BRAFi in melanoma [38,39]. However, whether CEP55 influences BRAFi resistance in melanoma by modulating the MAPK pathway remains unclear. To address this, we first assessed cell viability at various drug concentrations using the CCK8 assay in multiple melanoma cell lines, including A375, A2058, M14, SKMEL-28, and A875. The results showed that the A875 and A2058 cell lines were more resistant to vemurafenib (Fig. 6g). Based on these findings, we constructed CEP55 knockdown A875 cell lines (shCEP55-1/2 and NC) (Fig. 6h). Both A2058 and A875 melanoma cell lines were then treated with vemurafenib at increasing concentrations to determine their resistance to BRAFi. Our results revealed that CEP55 knockdown significantly reduced BRAFi resistance in both cell lines, with CEP55-deficient cells exhibiting greater sensitivity to BRAFi than NC cells. This effect was partially reversed by the addition of MAPK pathway activators (Fig. 6i,j).

### 3.9 High CEP55 Expression Indicates Better Response to Immunotherapy in Patients with Melanoma

**Figure 7:**
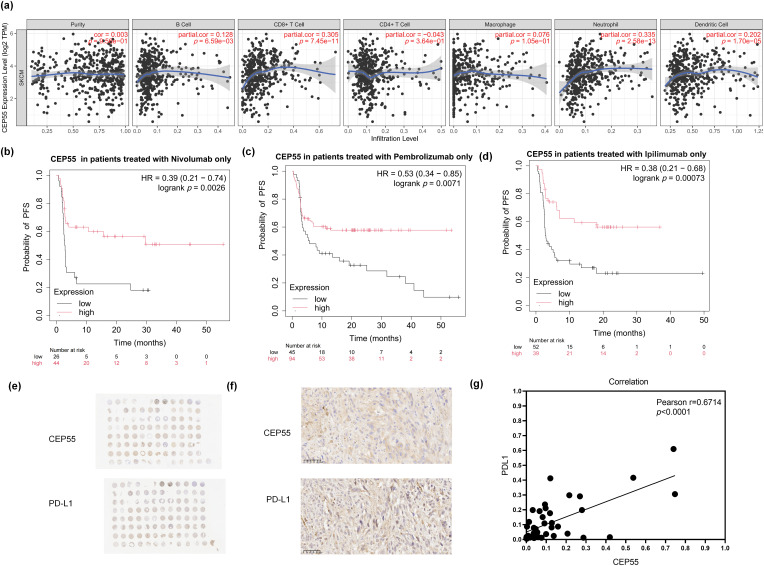
High CEP55 expression predicts better responses to immunotherapy in melanoma. **(a)** Relationship between CEP55 expression levels and immune cell infiltration in melanoma. **(b)** PFS of patients with melanoma treated with nivolumab alone, stratified according to CEP55 expression levels. **(c)** PFS of patients with melanoma treated with pembrolizumab alone, according to CEP55 expression levels. **(d)** PFS of patients with melanoma treated with ipilimumab alone, according to CEP55 expression levels. **(e, f)** IHC staining for CEP55 and PD-L1 in a melanoma tissue microarray, scale bar = 50 μm. **(g)** Positive correlation between CEP55 and PD-L1 expression levels in melanoma tissues

As RNA-seq results indicated that CEP55 is associated with B cells, IL-17, and other pathways related to immune response. We investigated its biological significance in melanoma immunotherapy. First, we analyzed the relationship between CEP55 and various immune cells in melanoma using the TIMER platform. Analysis of immune cell infiltration within the tumor microenvironment revealed a positive correlation between CEP55 expression and infiltration levels of B cells, CD8^+^ T cells, neutrophils, and dendritic cells. Conversely, CEP55 expression was not significantly correlated with the infiltration levels of other immune cells, such as CD4^+^ T cells (Fig. 7a). To further explore the relationship between CEP55 and immune checkpoints, we analyzed progression-free survival (PFS) in patients treated with nivolumab, pembrolizumab, or ipilimumab using the Kaplan-Meier Plotter platform. In the platform, there are 423 melanoma patients who received immunotherapy. We separately analyzed the data of patients treated with one of the above three drugs. Results showed that patients with high CEP55 expression had better PFS in all three groups (Fig. 7b–d). Additionally, we assessed the correlation between CEP55 and PD-L1 expression via IHC staining of tissue microarrays (Fig. 7e,f). The results demonstrated a positive correlation between CEP55 and PD-L1 expression (Fig. 7g). Collectively, these findings indicated that high CEP55 expression may predict a favorable response to immunotherapy in patients with melanoma and serve as a potential biomarker for immunotherapy response.

## 4 Discussion

Initially identified as a key regulator of cell division, the essential role of CEP55 in mammalian cell mitosis has been challenged by subsequent studies [40]. Numerous studies have demonstrated that alterations in CEP55 expression or mutations are associated with a spectrum of diseases, including a genetic disorder predominantly characterized by anencephaly [41]. Additionally, elevated CEP55 expression in diverse tumor types underscores its involvement in tumorigenesis, highlighting its potential as a tumor-promoting factor. Consequently, CEP55 has emerged as a promising prognostic indicator in various malignancies [11,26]. Previous studies have primarily focused on the effect of CEP55 on downstream PI3K/AKT signaling cascades, and the mechanism underlying CEP55-mediated activation of PI3K phosphorylation is well-documented [42–44]. Furthermore, studies have indicated that there is the possibility of interaction between CEP55 and the MAPK signaling pathway [16,37]. However, the role of CEP55 in AM onset and progression of AM remains unclear.

All tissue samples in this study were obtained from AM patients with high incidence in the Asian population, and all were untreated cases without targeted therapy or immunotherapy, verifying its abnormal expression in acral melanoma. Given the low response rate of this subtype to existing adjuvant treatment regimens, the abnormal expression of CEP55 may provide a new idea to reveal the progression mechanism in melanoma. So this study revealed a significant role of CEP55 in the pathogenesis of AM. Our study validated CEP55 overexpression in AM tumor tissues, revealing a relationship between CEP55 expression in tumor thickness and advanced TNM stage. These clinicogenomic correlations collectively indicated CEP55 as a promising biomarker with prognostic significance in AM. Our investigation demonstrated that CEP55 facilitated melanoma progression by promoting cellular proliferation, migration, and invasion, both *in vitro* and *in vivo*. RNA-seq analysis following CEP55 knockdown identified DEGs involved in MAPK pathway, immune responses, and other pathways, reflecting transcriptional regulation.

Interactions between CEP55 and epigenetics, has further elucidated the mechanisms underlying the role of CEP55 in tumors [20,45,46]. Despite these advances, the molecular mechanisms of CEP55 in melanoma remained poorly understood, necessitating further investigation. Our findings from RNA-seq and western blot analyses confirmed that CEP55 drived melanoma progression by activating the downstream MAPK signaling pathway. In addition, melanoma cells exhibited increased sensitivity to BRAFi after the CEP55 knockdown. In the rescue experiment, the concentration of vacquinol-1 we used referred to set according to the relevant literature, and preliminarily confirmed the relationship between CEP55 and MAPK [47,48]. However, the effect of drugs toxicity on cells may not be considered, and future research can further explore it. Studies have suggested that CEP55 is associated with adverse outcomes across various tumor types, potentially through its regulation of the cell cycle or by affecting exosomes [25,49,50]. And we found high expression of CEP55 may indicated a more favorable response to immune checkpoint inhibitors in patients, which indicated its role in regulating the tumor microenvironment [50]. CEP55 expression levels could become an essential indicator for predicting patient responses to immunotherapy [26,31,51].

This study identified CEP55 as a potential therapeutic target for melanoma, offering new insights into improving therapeutic responses and reducing drug resistance. Downregulation of CEP55 increased tumor sensitivity to BRAFi, highlighting its central role in the drug resistance of melanoma cells. In addition, elevated CEP55 expression was associated with improved responsiveness to immunotherapy in patients with melanoma, highlighting its potential as a critical biomarker for precision medicine approaches. These results suggested CEP55 expression status could stratify treatment approaches: For melanoma patients with low expression of CEP55, they may benefit from BRAFi/MEKi due to intact MAPK dependency. Otherwise, immunotherapy is more suitable for patients with high CEP55 expression. RNA-seq analysis revealed that CEP55 downregulation may lead to the suppression of the IL-17 signaling pathway, which may be one mechanism through which CEP55 participates in immune responses. IL-17 is a key factor in the immunotherapy response in melanoma [52]. A deeper understanding of the signaling pathways and molecular mechanisms regulated by CEP55 could facilitate the development of novel treatment strategies, thereby improving the efficacy and applicability of immunotherapy.

However, the specific underlying mechanisms remain unclear, likely because of the limited number of samples. In future studies, we will aim to explore the molecular mechanisms through which CEP55 regulates melanoma. Building upon our preliminary findings, future studies should include large-scale clinical trials involving patients with melanoma to evaluate whether CEP55 is a reliable predictor of response to immunotherapy. Considering the role of CEP55 in AM, further investigations could explore its function in other tumor types to assess its potential as a target gene across a broader range of malignancies, thus expanding the clinical relevance of this study.

## 5 Conclusion

The increased expression of CEP55 in melanoma correlates with poor prognosis. As a key regulator of melanoma cell proliferation, invasion, and migration, CEP55 represents a potential therapeutic target. High expression of CEP55 predicts a better response to immunotherapy in AM patients.

## Data Availability

The manuscript contains all essential data; data can be obtained from the corresponding authors upon formal request.
